# Re‐examination of statistical relationships between dietary fats and other risk factors, and cardiovascular disease, based on two crucial datasets

**DOI:** 10.1002/qub2.29

**Published:** 2024-01-22

**Authors:** Jiarui Ou, Le Zhang, Xiaoli Ru

**Affiliations:** ^1^ College of Computer Science Sichuan University Chengdu China; ^2^ Department of Gynecology and Obstetrics Beijing Chao‐Yang Hospital Capital Medical University Beijing China

**Keywords:** A/B test, bioinformatics, cardiovascular disease, nonparametric statistics

## Abstract

Cardiovascular disease (CVD) is the major cause of death in many regions around the world, and several of its risk factors might be linked to diets. To improve public health and the understanding of this topic, we look at the recent Minnesota Coronary Experiment (MCE) analysis that used *t*‐test and Cox model to evaluate CVD risks. However, these parametric methods might suffer from three problems: small sample size, right‐censored bias, and lack of long‐term evidence. To overcome the first of these challenges, we utilize a nonparametric permutation test to examine the relationship between dietary fats and serum total cholesterol. To address the second problem, we use a resampling‐based rank test to examine whether the serum total cholesterol level affects CVD deaths. For the third issue, we use some extra‐Framingham Heart Study (FHS) data with an A/B test to look for meta‐relationship between diets, risk factors, and CVD risks. We show that, firstly, the link between low saturated fat diets and reduction in serum total cholesterol is strong. Secondly, reducing serum total cholesterol does not robustly have an impact on CVD hazards in the diet group. Lastly, the A/B test result suggests a more complicated relationship regarding abnormal diastolic blood pressure ranges caused by diets and how these might affect the associative link between the cholesterol level and heart disease risks. This study not only helps us to deeply analyze the MCE data but also, in combination with the long‐term FHS data, reveals possible complex relationships behind diets, risk factors, and heart disease.

## 1 INTRODUCTION

Diet, especially fat intake, and its influence on cardiovascular diseases (CVD) has long been a key topic in the field of public health [1]. Many risk factors associated with CVDs could be influenced by dietary fats [2] and research shows that CVD has become the leading cause of death in many regions around the world [1]. For this reason, previous studies have focused on the three central questions. The first question asks whether there is any causality between replacing saturated fats with unsaturated fats and lowering the serum cholesterol. The second asks whether reducing cholesterol benefits CVD risks. The third question is regarding the meta‐relationship between, on one hand, dietary fats, risk factors, and, on the other hand, CVD as observed through convincing data [2]. A deeper statistical understanding of such relationships is crucial for improving public health regarding diets, influential risk factors, and heart diseases.

One of the most important diet‐heart studies is the Minnesota Coronary Experiment (MCE) directed by Ivan Frantz [3]. The experiment was a double‐blind randomized controlled dietary intervention trial conducted in 1968–1973, which aimed at examining possible causality between serum cholesterol and CVD by replacing common saturated fat with corn oil rich in *n*‐6 linoleic acid for the daily diet of patients in seven collaborating hospitals. Initially, the MCE involved 9570 adult participants, divided into control and intervention groups, where the only difference was the oil and fat sources of their diets [4]. Unfortunately, as later research [4] has pointed out, both Broste’s [5] thesis and Frantz’s [6] report on this experiment encounter the issue of selective reporting and lack of supplemental materials [4].

**FIGURE 1:**
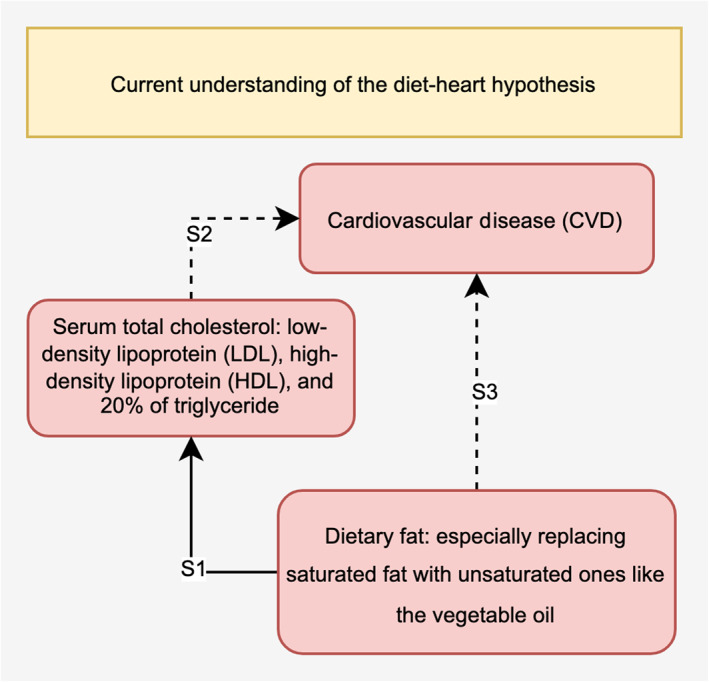
Diet‐heart hypothesis summary figure. The solid line represents commonly verified causality, while the dashed lines mean that causality is not yet established. S1 represents the relational test for Scenario 1, S2 represents the survival test for Scenario 2, and S3 represents the meta‐relationship analysis for Scenario 3 described later in the Methods section.

Thus, Ramsden et al. [4] collaborated with the National Institute of Health to recover as much original true data as possible from the MCE and then performed more comprehensive statistical analyses to test for the following three types of relationship, see also Figure 1.

1. Does replacing saturated fats with corn oil significantly reduce the serum total cholesterol level, which consists of low‐density lipoprotein (LDL), high‐density lipoprotein (HDL), and 20% of the total triglyceride level [2, 3, 4]?
2. Does reducing the serum total cholesterol level significantly affect the CVD hazard rates?
3. Are there significant links between low saturated fat diets, cholesterol levels, and CVDs?

Three scenarios exist. These are: (a) verify the statistical causality between conducting low saturated fat diets and reducing the serum total cholesterol level; (b) examine statistical relationships between lowering the serum total cholesterol and CVD mortalities; and (c) look for meta‐relationship and influencing factors in how diets affect CVD events. The above scenarios are represented by S1, S2, and S3 in Figure 1, respectively. These three scenarios represent the current typical understanding and statistical study objects regarding how changing a person’s dietary fat intake can influence crucial body indices [4], including cholesterol and blood pressure levels, and whether these really affect risk of CVD events [4]. Ramsden’s research greatly raised public interest about the diet‐heart disease hypothesis and brought part of the crucial MCE summary data onto the stage [4], but each of the 3 relational tests that Ramsden conducted still suffers from potential statistical problems [7].

The first analysis Ramsden [4] did was a commonly used *t*‐test regarding the causality between corn oil intervention diet and reductions in the serum cholesterol level. He utilized the partially recovered data from MCE and derived a *p*‐value of <0.01 for control versus intervention groups. However, note that the normal distribution assumption [8, 9, 10, 11, 12] of the experiment data was not validated, plus the recovered sample size was relatively small compared to typical observational studies; the appropriateness of using paired *t*‐test might be questionable as some of the requirements can be violated. Therefore, our first challenge in this work is to test the causality hypothesis between corn oil diets and serum cholesterol levels in a more generalized sense when normality and sample size assumptions do not necessarily hold.

Furthermore, the second analysis in Ramsden’s [4] publication implemented a Cox regression model [13, 14, 15] for survival analysis to test for the relationship between cholesterol level reduction and risks of death. The recovered data was split into two groups depending on whether the participant’s cholesterol level decreased at the end of the MCE. Then, Ramsden conducted a multivariate Cox regression and concluded that, though reducing the LDL level is traditionally believed to lower the risk of CVDs, the MCE results revealed no mortality benefit in the overall cohort when looking at the serum total cholesterol adding up together [4, 5]. Nevertheless, the data that Ramsden used for calculating the hazard ratio was deaths from all‐causes within the recovered sample, rather than deaths caused by CVDs only. Moreover, several studies show that the recovered MCE sample might suffer from small sample size and right‐censored bias where the majority of the patients do not have follow‐up mortality measurements [7]. Hence, the Cox model that requires a large unbiased sample is not quite effective nor appropriate for conclusions regarding the relationships between serum total cholesterol and CVD risks [7]. Thus, the inferred relationship from this survival analysis might be challenged [7]. For these reasons, our second challenge is to test the relationship hypothesis between reducing serum total cholesterol and its effects on CVD hazards when the sample is small and right‐censored.

Lastly, the third analysis by Ramsden [4] is a meta‐analysis of the links between unsaturated fat diets, other risk factors such as cholesterol and CVD risks. He combined the recovered MCE research with five other similar randomized controlled trials to conclude that the low saturated fat diet does not significantly benefit total‐cholesterol related CVD risks [4]. Additionally, he later discussed the limitations and inconsistencies in some of other observational studies on the diet–heart disease relationship and why he did not use such long‐term data [16]. However, according to other research, observational studies that include possible confounding factors from long‐term measurements are also important because all the short‐term randomized controlled trials suffer from sample bias and follow‐up measurements issues [7]. In addition, development of any CVDs is a biologically complicated chain reaction in the body, so large population data with several risk factors measured from cohort studies can be preferable [17]. For instance, hypertension caused by diet might also influence disease risks [17]. Thus, our third challenge in this work is to test the links between diets, risk factors, and CVD risks when the long‐term observational data are considered and when the parametric assumptions do not hold.

To solve the shortcomings of previous studies, this research proposes three innovative schemes:

First, when *t*‐test assumptions do not hold, we implement a nonparametric permutation test [9, 10, 11, 18] where our principal test statistic is the absolute difference in mean percentage change of cholesterol between the control and intervention group.

Second, we estimate the hazards by observing CVD‐caused deaths through a typical rank estimating process [19] in each group, and then perform a resampling‐based hypothesis test to examine the association between reducing serum total cholesterol and the effects on CVD hazards when the sample is small and right‐censored.

Third, we expand the data with the long‐term observational Framingham Heart Study (FHS) data and then performed an A/B test [9, 10, 11, 12, 18, 19, 20, 21, 22], hypothesis testing regarding relationships between diets, risk factors including cholesterol and blood pressure range, and CVD risks when the observational data is considered.

To summarize, this study aims to re‐examine the statistical relationships between dietary fat intakes, other potential risk factors, and CVD risks. According to our results, the nonparametric permutation test suggests that the causality between low saturated fat diets and reducing serum total cholesterol is statistically obvious. However, reducing the serum total cholesterol through low saturated fat diets does not significantly influence CVD mortality in the diet group of the MCE intervention. Lastly, with the expanded observational study data, our A/B test result suggests a more complicated conclusion that, in the relationships between diets, risk factors, and heart diseases, abnormal blood pressure ranges might confound with the serum total cholesterol level and thus affect how the cholesterol associates with CVD risks.

## 2 RESULTS

### 2.1 Scenario 1: Hypothesis testing using the nonparametric permutation test

**FIGURE 2:**
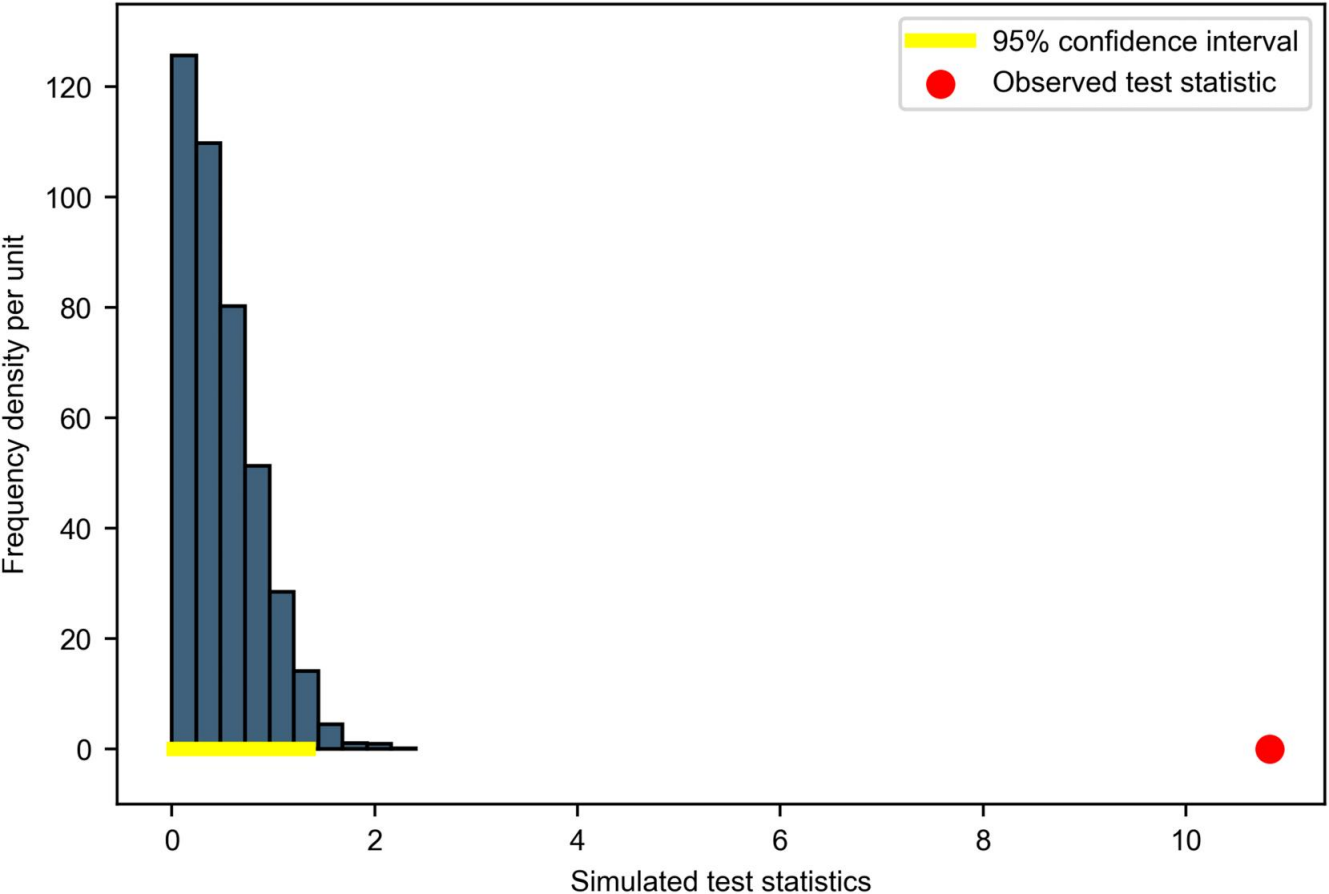
Permutation test results from Scenario 1.

Figure 2 shows that most simulated test statistics, represented by the bins, lie in the range between 0 and 2, as indicated by the yellow line through the permutation test from Scenario 1 using Ramsden’s [4] sample. Here, the *x*‐axis represents the value of each simulated test statistic, and the *y*‐axis represents the frequency density per unit. The red dot at the value around 13 indicates the true observed test statistic of all the 2355 samples from Ramsden’s [4] study. Hence, as the observed value sits far away from most simulated values, our empirical *p*‐value is then <0.014, suggesting that we should reject the null hypothesis. Thus, we consider that low saturated fat diets are most likely to reduce the serum total cholesterol, especially the LDL level, compared to that in normal diets.

### 2.2 Scenario 2: Association examination using the resampling‐based rank test

**FIGURE 3:**
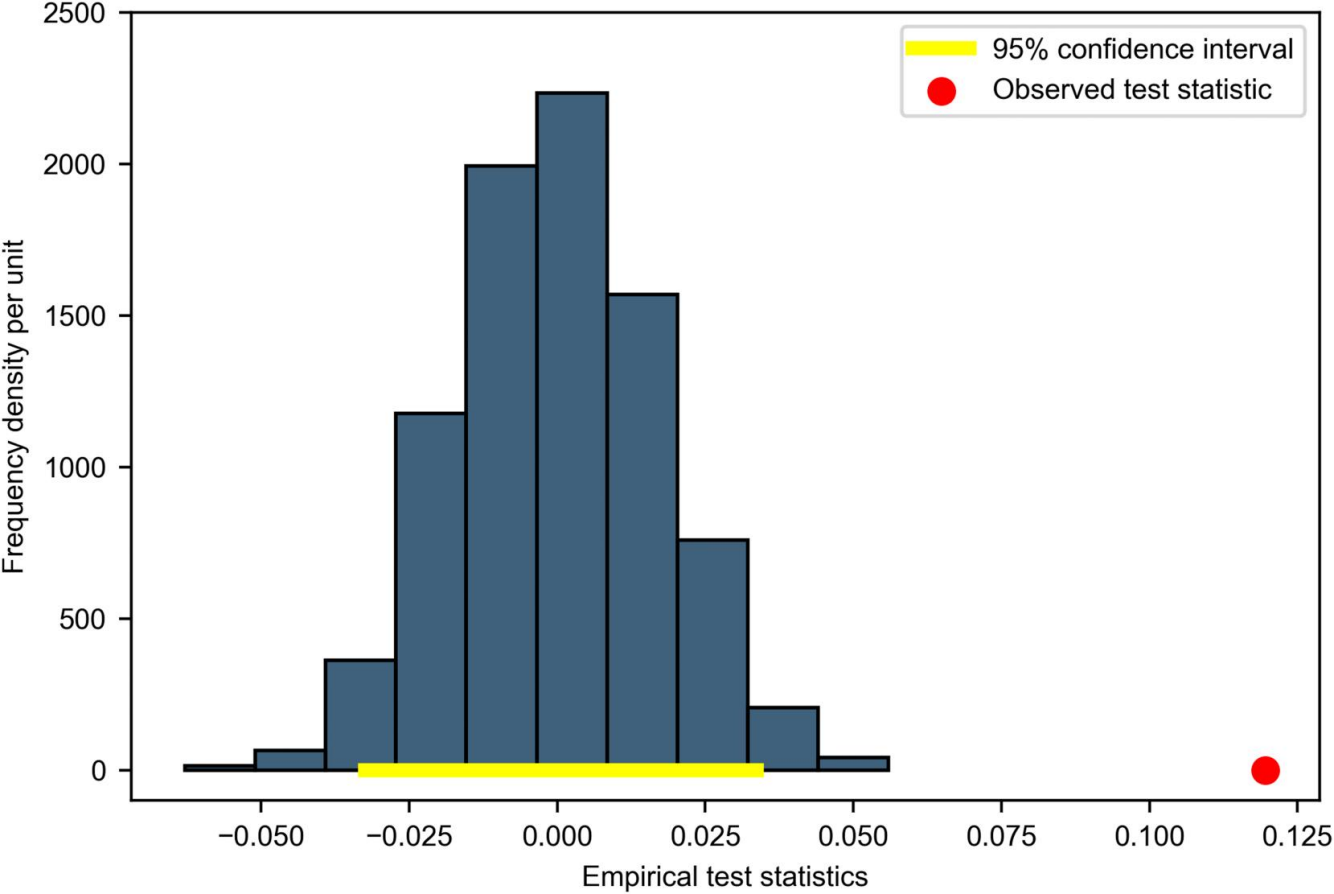
Control group results from Scenario 2.

**FIGURE 4:**
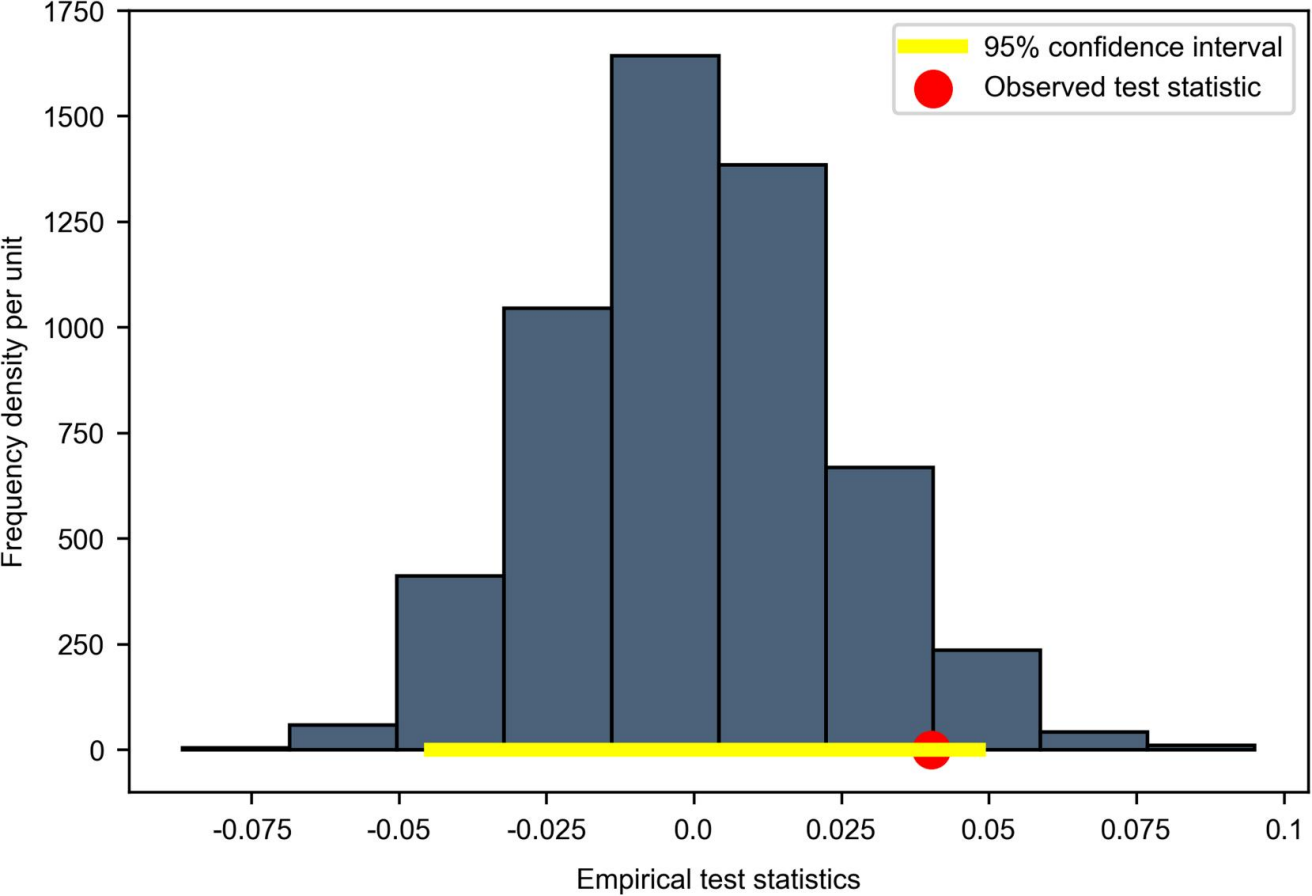
Diet group results from Scenario 2.

As Ramsden et al. [4] did, we first split the control and the diet groups, then further divide each group into two subgroups of the increasing cholesterol group versus the decreasing cholesterol group. Resampling rank test results from Scenario 2 are shown in Figures 3 and 4, where the *x*‐axis represents the value of empirical test statistics at each resampling iteration, and the *y*‐axis represents the frequency density per unit.

Figure 3 shows that, for the control group, most of our test statistics values from the resampling rank test distribute normally between −0.03 and 0.03 as represented by the bins, while the true observed hazards difference between the two subgroups, indicated by the red dot, is around 0.12 from Scenario 2 based on Ramsden’s [4] sample. Hence, as the true observed value is separated from most empirical test statistics, we reject the null hypothesis that increasing or reducing serum cholesterol level does not affect the CVD‐caused hazard ratio. This means that for the control group, change in cholesterol level seems to affect CVD hazards.

However, for the diet group, Figure 4 shows that most of our test statistics values lie in the interval from −0.05 to 0.05 as represented by the bins, but the true observed hazards difference is roughly 0.04 as indicated by the red dot, which settles inside the above interval. In this case, our precise empirical *p*‐value turns out to be around 0.067, which is greater than the significance level of 0.05. Hence, as the true observed value does not significantly deviate from most of our empirical values under the null hypothesis, we fail to reject the null hypothesis. Interestingly, this means that, for the diet group, we fail to prove that lowering cholesterol levels influences CVD hazards.

In terms of statistical power and performance comparison, the empirical power of such bootstrap‐based rank test has been proved in Ditzhaus’ work [23] to be similar to or even outperform typical survival models such as the Cox model and the Gill–Schumacher test for biased right‐censored dataset [23].

### 2.3 Scenario 3: A/B test with long‐term FHS data

To begin with, our calculated correlation coefficient between the cholesterol level and the diastolic blood pressure from Equation (4) is around positive 0.33, suggesting that they may weakly confound with each other. Hence, we continue the process of A/B test as shown in Figure 2C.

**FIGURE 5:**
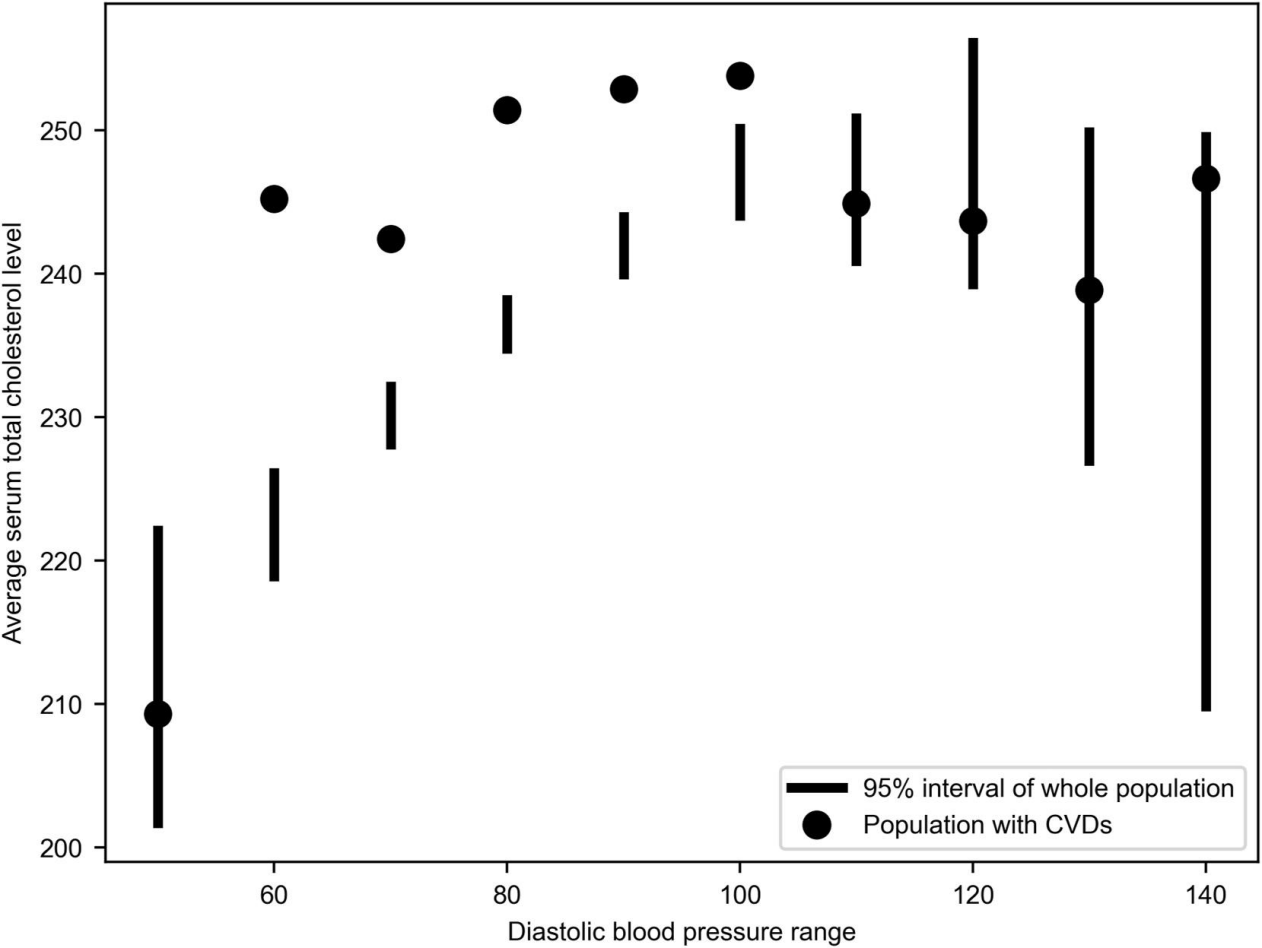
Observational study results from Scenario 3.

Figure 5 demonstrates that the dots settle outside the confidence interval for average serum total cholesterol levels when the diastolic blood pressure is between 60 and 100 mmHg but those dots lie inside the interval for more abnormal blood pressure ranges. In this figure, lines indicate the 95% confidence interval of the average cholesterol in the whole population at the current blood pressure range. Dots indicate the observed average cholesterol level of those who have CVDs in the current population sample. Thus, one crucial finding is that, caused by diet, when the diastolic blood pressure is above 100 mmHg or below 60 mmHg, the association rule of risk factors and CVD risks starts to change. Specifically, when the diastolic blood pressure is above 100 or below 60 mmHg, the serum total cholesterol level of those who have CVDs is not statistically significantly different from those who do not; thus, the serum total cholesterol level does not seem to be associated with CVD for this group of people. But for diastolic blood pressure ranges between 60 and 100 mmHg through regular diets, the cholesterol level of those who have CVD deviates a lot from those who do not; hence, the cholesterol level is likely to be associated with CVDs for people with diastolic blood pressure in this range.

## 3 DISCUSSIONS

In this study, we aim to re‐examine the statistical relationships between dietary fats, other risk factors, and heart disease risks, based on previous studies [4] in the following three scenarios.

First, using the recovered MCE data [4] from Ramsden’s [4] research, we develop a nonparametric permutation test for examining the causality between low saturated fat diets and reduction in cholesterol. Since the sample data is small‐sized, the nonparametric test should be more appropriate than the *t*‐test that Ramsden [4] originally used. Our permutation test result with a low nonparametric *p*‐value <0.014 indeed agrees with this idea and further validates that low saturated diets can cause reductions in the serum total cholesterol level.

Second, as suggested by various studies [7, 15, 19] the Cox model that Ramsden [4] used based on such a small‐sized right‐censored dataset is not precisely reliable. Hence, to properly fit such data, we implement a resampling‐based rank test using estimated CVD hazards to test the hypothesis that reducing serum total cholesterol significantly impact CVD deaths. Our rank test result, by resampling, shows that the observed estimated hazards of the diet group do not deviate a lot from most of our empirical test statistics. Thus, this relational hypothesis does not necessarily hold for the diet group, raising questions to Ramsden’s [4] conclusions.

Third, we investigate the relationships between diets, other risk factors, and CVD risks using the A/B test in which the long‐term observational data is considered. With more risk factors involved, our A/B test allows for controlling one variation at a time to test for more detailed relationships. The result in Section 2.3 indeed shows a more complicated relationship that when the diastolic blood pressure is above 100 or below 60 mmHg, the cholesterol level seems not to be associated with CVD risks anymore, but when the blood pressure is between 60 and 100 mmHg, the associative link of the cholesterol to CVDs is still present.

Although we attempt to perform a statistical re‐examination of relationships between dietary fats, other risk factors, and CVD risks under three scenarios with a 0.05 significance level, there are still limitations to be considered. For instance, our nonparametric methods tend to be computationally expensive, meaning that they may be inefficient if we include large datasets [23]. Hence, it is difficult to generalize if other complicated datasets are considered, the efficiency of tests could be very low [23]. Moreover, even with a huge amount of resampling, permutation tests may turn out to have significant Type‐I errors and less power when dealing with some small sample cases [23]. In our case, considering our dataset with the MCE data and the long‐term data, statistical calculations were made to allow greater than 95% statistical power, which is correlated to the significance level and effective sample size of the data [24]. However, for other extreme datasets, to improve validity of conclusions, we should develop more effective and comprehensive nonparametric or semi‐parametric methods to accommodate more complex data for meta‐analyses [24]. In the distant future, with a convincible totality of the evidence, we should aim to establish strong statistical links between diets, various risk factors, and CVD across different regions around the world.

## 4 CONCLUSIONS

To conclude, the contribution of this study is three‐fold: (a) For the “S1” link in Figure 1, our nonparametric permutation test with the MCE sample data validates the causal link between unsaturated fat diets and reducing cholesterol level. (b) For the “S2” link in Figure 1, reductions in the serum total cholesterol level does not strongly affect CVD‐caused deaths in the diet group based on the resampling‐based rank test using the small‐sized right‐censored MCE data. (c) Combining the “S1” and “S2” links together, for the “S3” meta‐relationship in Figure 1, our A/B test results with expanded long‐term FHS observational data further show that relationships between dietary fats, other risk factors, and CVD risks are very complicated as factors such as blood pressure ranges with abnormality caused by diet can statistically impact the interaction between the cholesterol level and CVD risks.

## 5 MATERIALS AND METHODS

### 5.1 Data sources

Our two main data sources are as follows: firstly, the materials in Ramsden’s publication [4] containing a small‐sized recovered dataset of the MCE study; secondly, the FHS observational dataset that includes more populations and risk factors [25]. In the MCE dataset, there were 1179 samples in the diet group and 1176 samples in the control group, adding up to 2355 participants in total [8]. For each participant, attributes including age, diet conditions, CVD conditions, and serum cholesterol levels were recorded during the MCE study [8]. We further summarize MCE dataset and use it for analysis in Scenario 1 and 2. On the other hand, the FHS dataset used in Scenario 3 has 3840 samples in total. It has information about age, cholesterol levels, diastolic blood pressure, and CVD conditions for each participant [2].

### 5.2 Computational methods

As indicated in Figure 1, each scenario has distinct tasks and aims for different hypotheses. From the “S1” link in Scenario 1, the permutation test is a great substitute of *t*‐test since it does not assume any prior distributions of the sample and it has adequate performance for deriving empirical *p*‐value in such scenario [23]. From there, the “S2” link in Scenario 2 is suitable for a resampling‐based testing method to help with simulation with the right‐censored data and a rank‐based method for dealing with data from unknown distribution [23]. Finally, combining “S1” and “S2” together with extra long‐term data, the “S3” link in Scenario 3 can be examined using the A/B test, which exactly does the job of testing the statistical influence of multiple variants instead of two variables. Thus, we implement 3 specific tests for our 3 scenarios to address each challenge appropriately.

For the first scenario, we aim to test whether there is a significant difference between the control and diet group when the normal distribution and equal variance assumptions of using *t*‐test are possibly violated. Indeed, as we explore the basic statistics of the recovered sample, the standard deviation relating to cholesterol change in the diet group is 13%, which is smaller than 16% in the control group. In addition, the group sizes are relatively small and unequal, while the normal distribution of the variable cannot be well verified. Hence, the appropriateness of using parametric *t*‐test is questionable [23]. In this case, the nonparametric permutation test is a great substitute since it does not assume any prior distributions or equal variance of the sample and it has adequate performance for deriving empirical *p*‐value using the resampling technique [23].

For the second scenario, the situation is a bit different since we are dealing with hazards among different groups using the small‐sized right‐censored dataset to seek for relationships between reducing serum total cholesterol and CVD‐caused deaths. As stated in Ditzhaus’ work on bootstrap rank tests, actual performance of the Cox model under small right‐censored sample is concerning [23]. In this case, resampling is a useful method to help with simulation and rank‐based methods are suitable for dealing with hypothesis testing related to hazards [23]. Hence, regarding the second hypothesis, to better fit the small‐sized right‐censored data, we utilize a bootstrap rank test instead of the Cox model for survival analysis.

**FIGURE 6:**
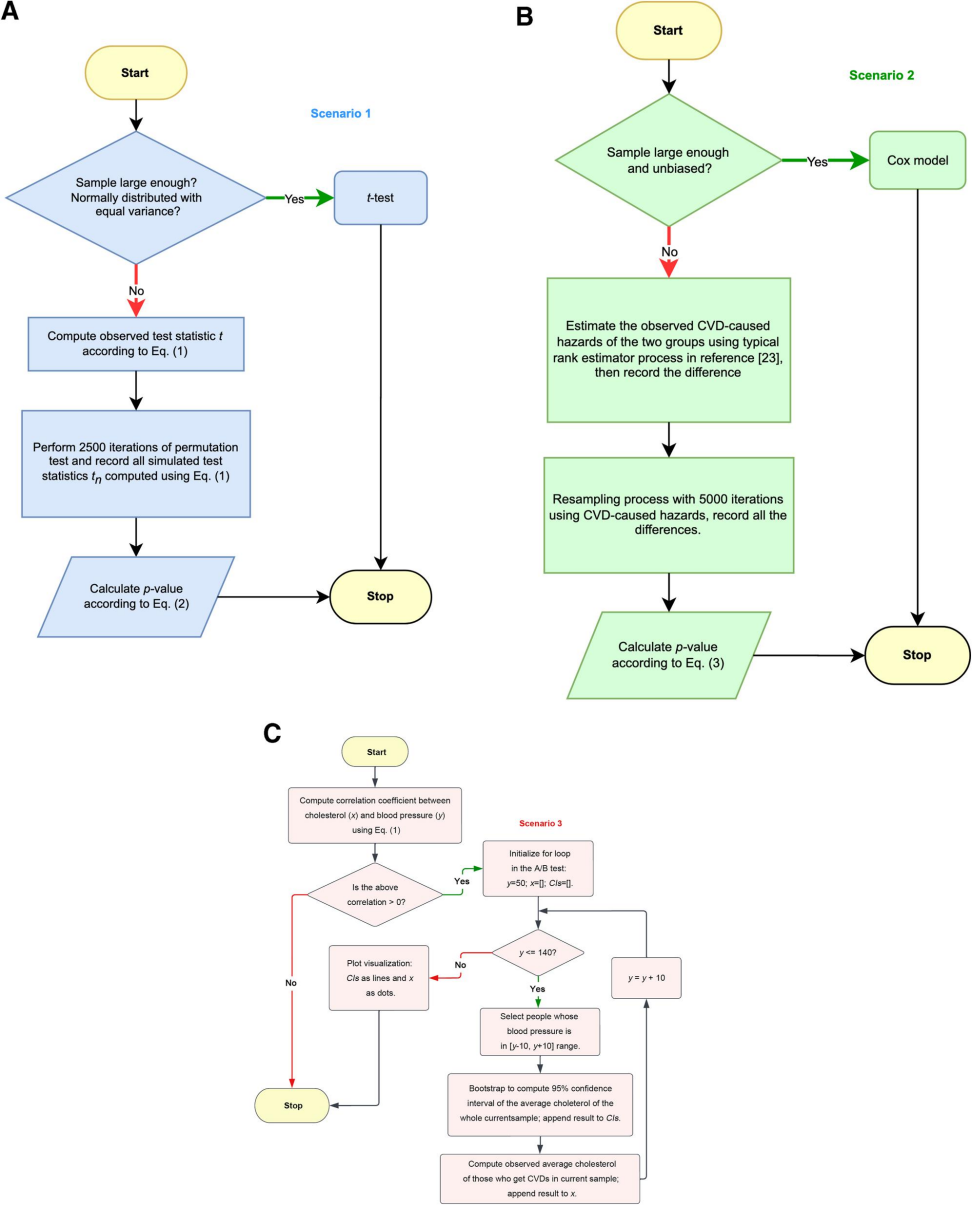
Overall workflow of this study consisted of three parts. (A) Scenario 1, permutation test based on the MCE data. (B) Scenario 2, the resampling‐based test using the MCE data. (C) Scenario 3, A/B test based on FHS data. In the for‐loop, *y* refers to discretized diastolic blood pressures, *x* refers to average serum total cholesterol levels, and *CIs* refers to the set of 95% confidence interval results.

Lastly, for the third scenario, different from the previous scenarios, our purpose is to test for statistical relationships among different factors with the augmented data. Thus, the A/B test does exactly the job of testing the statistical influence of multiple variants [23]. Hence, the overall procedure is divided into three parts shown as in Figure 6: the first part is to draw conclusions on the causality between low saturated fat diets and reduction in serum total cholesterol using the MCE sample to answer the part A link in Figure 1; the second part is to examine the relationship between reduction in the serum total cholesterol and CVD‐caused hazards with the small‐sized right‐censored data to answer the part B link in Figure 1; the third part is to augment our dataset with the FHS data and to further test the statistical correlation and association between diets, risk factors, and CVD risks when the long‐term data is considered, to draw conclusions on the part C link in Figure 1. Combining the conclusion from all three scenarios, we then finally discuss the meta‐relationships between dietary fats, risk factors, and CVDs.

#### 5.2.1 Nonparametric permutation test for testing the first hypothesis

For the first question, to solve the potential problem of violating the paired normality and large sample size assumptions, we implement a nonparametric permutation test to perform the hypothesis testing when the sample is small.

For the permutation test, refer to Figure 6A, the null hypothesis is set as: a low saturated fat diet does not affect the serum total cholesterol level, versus the alternative hypothesis: a low saturated fat diet significantly reduces the serum total cholesterol level. Compared to the *t*‐test, as we do not make any prior assumptions on the distribution, we can now empirically calculate the test statistic to perform a permutation test with 2500 rounds of permutation. Let *t* denote our observed test statistic, let *d*_*i*_ denote the *i*th percent change of cholesterol level in the diet group and let *c*_*j*_ denote the *j*th percent change of cholesterol level in the control group. Then, the test statistic can be calculated using Equation (1):

(1) $$t=\left|\frac{\sum_{i=1}^{1179}d_{i}}{1179}-\frac{\sum_{j=1}^{1176}c_{j}}{1176}\right|$$

Now, at the *n*th iteration, we first randomly permute the sample and then calculate the *n*th simulated test statistic *t*_*n*_ using the permuted sample according to Equation (1). After 5000 iterations, we can terminate the process and derive our nonparametric *p*‐value according to Equation (2), where {…} denotes the mathematical indicator function:

(2) $$p\text{‐value}=\frac{1}{2500}\sum_{n=1}^{2500}1{\{t}_{n}⩾t\}$$

#### 5.2.2 Resampling‐based rank test based on estimated CVD hazards

For the second part, instead of employing deaths of all causes, we only use the death events caused by CVDs in each group. When the sample is small‐sized and right‐censored, the resampling‐based rank test may be more effective [19]. Inspired by this, we first estimate the observed hazard rate through the typical rank estimator process [8, 11, 26] using CVD‐caused deaths only, and then perform a resampling process for hypothesis testing as shown in Figure 6B. We, finally, compute the resampling *p*‐value according to Equation (3). We set the null hypothesis as: raising or reducing serum total cholesterol level does not affect the CVD‐caused hazard ratio, versus the alternative hypothesis: reducing serum total cholesterol level significantly affects CVD‐caused hazard ratios. If we denote *h* as the observed difference in the estimated hazards between the two groups, and denote *h*_*n*_ as the *n*th simulated difference in the 5000 iterations of bootstrapping, then our two‐tailed resampling *p*‐value can be calculated using Equation (3):

(3) $$p\text{‐value}=\frac{1}{5000}\sum_{n=1}^{5000}1\left\{\left|h_{n}\right|⩾\left|h\right|\right\}$$

#### 5.2.3 A/B test with more factors based on the FHS data

As for the third challenge of the meta‐relationship between diets, influential risk factors, and CVD risks, to expand the dataset and take long‐term observations into account, we extract the first wave of data from the FHS [25], which is a crucial long‐term observational study about diet and heart disease. In this dataset, another influential risk factor, namely diastolic blood pressure, is included along with the serum total cholesterol so that we can test for more comprehensive relationships between diet, other risk factors, and CVD risks [26]. Since, after expanding our data, the large sample does not necessarily meet the parametric test assumptions, we then implement the A/B test as shown in Figure 6C to examine for correlation and association relationships. We compute the Pearson correlation coefficient defined by Equation (4):

(4) $$\mathrm{corr}\left(x,y\right)=\frac{1}{n-1}\sum_{i=1}^{n}\left(\frac{x_{i}-\bar{x}}{s_{x}}\right)\left(\frac{y_{i}-\bar{y}}{s_{y}}\right)$$

Here, *x* and *y* are the sample cholesterol level and the blood pressure respectively; *n* denotes our sample size; $\bar{x}$ and $\bar{y}$ represent the average cholesterol and blood pressure of the sample; *s*_*x*_ and *s*_*y*_ represent the standard score of cholesterol and blood pressure respectively. Then, if any correlation is presented, we now test for the relationships between diets, risk factors including diastolic blood pressure, serum total cholesterol, and CVD risks through the bootstrap process of our A/B test described in Figure 6C. We can then derive a visualization result regarding influences of the blood pressure with cholesterol on CVD risks.

## AUTHOR CONTRIBUTIONS

**Jiarui Ou**: Conceptualization; data curation; formal analysis; investigation; methodology; visualization; writing – original draft; writing – review & editing. **Le Zhang**: Conceptualization; funding acquisition; investigation; methodology; project administration; resources; supervision; validation; writing – original draft; writing – review & editing. **Xiaoli Ru**: Conceptualization; methodology; resources, validation; writing – review & editing.

## CONFLICT OF INTEREST STATEMENT

The authors Jiarui Ou, Le Zhang and Xiaoli Ru declare that they have no conflict of interest or financial conflicts to disclose.

## ETHICS STATEMENT

All procedures performed in studies involving animals were in accordance with the ethical standards of the institution or practice at which the studies were conducted, and with the 1964 Helsinki declaration and its later amendments or comparable ethical standards.
